# P-614. Acceptance of vaccination - strategies for populations of Ukrainian children migrating to Poland

**DOI:** 10.1093/ofid/ofae631.812

**Published:** 2025-01-29

**Authors:** Anna Dzielska, Dorota Kleszczewska, Katarzyna Lewtak, Joanna Mazur

**Affiliations:** Institute of Mother and Child, Warsaw, Mazowieckie, Poland; Institute of Mother and Child Foundation, Warsaw, Poland, Warsaw, Mazowieckie, Poland; Medical University of Warsaw, Warsaw, Mazowieckie, Poland; University of Zielona Góra, Collegium Medicum, Zielona Gora, Lubuskie, Poland

## Abstract

**Background:**

The presence of a significant number of refugees from Ukraine in Poland creates a need to understand and improve the immunisation status of children in this population. UNICEF and WHO data indicate potential challenges in maintaining adequate vaccination coverage in the refugee population. In this context, this study aims to identify factors influencing vaccination decisions among parents of Ukrainian children living in Poland. The main objective of this project is to increase vaccination coverage among Ukrainian children (0-5 years) in Poland.

**Methods:**

A pilot study to identify the main barriers and determinants of vaccination decisions among parents of newly arrived war migrants from Ukraine was conducted online and included a group of 187 refugees from Ukraine, most of whom were women. Participants in the study were analysed in terms of their barriers and concerns about vaccinating their children.

**Results:**

Preliminary results of the survey indicate that the main sources of information about vaccination for Ukrainian parents are doctors and the media. The most commonly reported barriers include difficulties in accessing health services, lack of knowledge about vaccination schedules, and language barriers. Concerns about returning to Ukraine and lack of information about the effects of vaccines are also important. Based on the findings, a behaviour change strategy based on the COM-B theoretical model and a systems approach is proposed. The implemented education and outreach activities, supported by media campaigns and psychosocial support, should aim to increase the capability, motivation and opportunity of Ukrainian parents to make informed decisions about vaccination. Between 24.02.2022 and 31.01.2024, 37,315 Ukrainian children aged 0-5 years were vaccinated in Poland.

**Conclusion:**

The effectiveness of interventions should be further investigated; however, the available evidence suggests that a comprehensive approach involving a variety of systems can contribute to improving the immunisation status of children among the Ukrainian refugee population in Poland.

**Disclosures:**

**All Authors**: No reported disclosures

